# *Helicobacter pylori* infection leads to KLF4 inactivation in gastric cancer through a TET1‐mediated DNA methylation mechanism

**DOI:** 10.1002/cam4.2892

**Published:** 2020-02-04

**Authors:** Rongrong Zhao, Zhengxia Liu, Wenting Xu, Le Song, Haifeng Ren, Yang Ou, Yakun Liu, Siying Wang

**Affiliations:** ^1^ Department of Physiopathology Anhui Medical University Hefei Anhui China; ^2^ Department of Pathology First Affiliated Hospital of Anhui Medical University Hefei Anhui China

**Keywords:** CagA, gastric cancer, *H pylori*, KLF4, TET1

## Abstract

Krüppel‐like factor 4 (KLF4) has a tumor suppressor role in the progression of gastric cancer (GC), and inhibition or loss of KLF4 expression was identified in GC. The aim of this study was to explore the new molecular mechanism of KLF4 inactivation in gastric cancer. Herein, we report that *Helicobacter pylori* infection or Cag pathogenicity island protein A (CagA) gene transduction resulted in KLF4 expression downregulation and promoted gastric epithelial cell and gastric cancel cell proliferation, migration, and colony formation. Mechanistically, we found that CagA gene transduction led to DNA methylation of the KLF4 promoter, an effect that was relevant to the significant downregulation of TET1 expression. Causally, knockdown of TET1 expression decreased KLF4 expression, whereas overexpression of TET1 had the opposite effect. Clinically, we found that KLF4 expression and the 5‐hmC levels were lower in GC cells with *H pylori* infection than in GC cells without *H pylori* infection. Thus, our study not only sheds new light on how *H pylori* infection promotes the progression of GC but also elucidates a novel mechanism of KLF4 inactivation in GC pathogenesis. During pathogenesis, an alteration in the *H pylori*/CagA‐TET1‐KLF4 signaling pathway plays a critical role, suggesting that this pathway may be a prospective target for gastric carcinoma intervention and therapy.

## Graphical abstract

CagA gene transduction increases KLF4 promoter methylation level, which leads to inactivation of KLF4. Helicobacter pylori infection leads to KLF4 inactivation in gastric cancer through a TET1‐mediated DNA methylation mechanism.

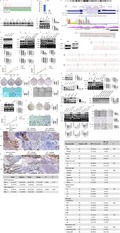

## 1 INTRODUCTION

Gastric cancer (GC) originates from the gastric mucosal epithelium, is a common malignant tumor, and has a mortality rate that is ranked second for tumor‐related diseases.^1^, ^2^ Surgery is currently the most effective treatment for GC, but targeted therapy is still the main treatment for patients who cannot tolerate surgery or for patients with recurrence after surgery.^3^ Targeted therapies, such as ramucirumab (an anti‐VEGFR‐2 antibody) and trastuzumab (an anti‐HER2 antibody [also known as ERBB2]), were used to treat cancer patients in the clinic.^4^ With the development of precision medical treatment and differences in patient tolerance, it is urgent to find effective therapeutic targets for GC and to research the underlying mechanism.

Krüppel‐like factor 4 (KLF4) is expressed in terminally differentiated epithelial cells of the gastrointestinal tract, lungs, and several other tissues.^5^ The function of KLF4 is mainly to regulate cell differentiation and apoptosis.^6^ It has been proposed that promoter methylation is required for KLF4 inactivation in GC^6^; clinical trials have demonstrated that targeting specific molecules to activate KLF4 is beneficial for therapeutic intervention of advanced solid tumors.^7^ However, the molecular mechanisms of KLF4 need to be explored in GC.

*Helicobacter pylori* infection is a risk factor in GC tumorigenesis and other gastric malignancies.^8^ However, strains of *H pylori* carrying the cytotoxin‐associated antigen A (CagA) gene are related to gastric carcinoma.^9^ CagA, the most important virulence factor of *H pylori*, enters into gastric epithelial cells with the help of the T4SS system and influences the expression of tumor suppressor genes in the host, such as GKN1, RUNX3, and P53.^10^, ^11^, ^12^, ^13^ We have found that *H pylori* CagA promotes malignant transformation of gastric epithelial cells through the dysregulation of mir‐155/KLF4 signaling pathway.^14^ So, is there any other way that *H pylori*/CagA affects KLF4 expression?

According to a previous report, promoter methylation is closely related to the inactivation of KLF4.^6^ In addition, ten‐eleven translocation (TET) enzymes mainly oxidize 5‐methylcytosine (5‐mC) into 5‐hydroxymethyl cytosine (5‐hmC) and participate in cytosine methylation and cell differentiation.^15^, ^16^, ^17^ However, it has been reported that TET1 decreased prominently in CagA‐infected GC tissues compared with that in para‐carcinoma tissues.^18^ Hence, we sought to explore how KLF4 is inactivated in GC and aimed to analyze the relationships among CagA, KLF4, and TET1.

## 2 MATERIALS AND METHODS

### 2.1 Reagents and cell culture

The GES‐1 cell line was provided by the Shanghai Institute of Cell Biology, Chinese Academy of Sciences. The AGS, SGC‐7901, and SK‐GT5 cell lines were purchased from ATCC. SGC‐7901 and SK‐GT5 cells were cultivated in Dulbecco's modified Eagle's medium (DMEM) and an environment of 5% CO_2_ at 37°C, and GES‐1 cells were cultivated in Roswell Park Memorial Institute 1640 (RPMI 1640) medium supplemented with 10% fetal bovine serum (Life Technologies/Gibco), 100 U/mL penicillin, and 100 µg/mL streptomycin (Life Technologies/Gibco). The plasmids of αSR and CagA‐wild type were purchased from Dr Masanori Hatakeyama of Japan's Tokyo University. The pEGFP‐N1 plasmid was purchased from Clontech, the plasmids TET1‐wt and pX459 were obtained from Addgene, and pX459‐TET1‐sgRNA was constructed in the laboratory. The liposome transfection reagent Lipofectamine 3000 was purchased from Invitrogen in the United States. Antibodies against HA were purchased from OriGene, antibodies against CagA were purchased from Santa Cruz, antibodies against 5‐hmC were purchased from Active Motif, and antibodies against KLF4 and TET1 were purchased from Santa Cruz.

### 2.2 Cell coculture with bacteria

GES‐1 cells were seeded into a six‐well plate, and the *H pylori* showed needle‐like, moist, colorless, and translucent colonies. The lawn was found in the place where the inoculum was more abundant. It was placed in a 10% CO_2_, 85% N2, and cultured in a 37°C incubator. *H pylori* precipitate was collected when the cells adhered. Then, sterile PBS was used to dilute the bacterial suspension, and a spectrophotometer was used to measure the bacterial concentration, where 1 OD = 1 × 10 6608 CFU/mL with different concentrations of *H pylori* suspension. The number of bacteria per cell was divided among the following groups: the control group (without *H pylori*), 25:1 experimental group, 50:1 experimental group, 100:1 experimental group, and 150:1 experimental group. Cultures were kept in an incubator at 37°C and 5% CO_2_, the supernatant was removed after 48 hours, and the cells were washed with PBS three times and lysed using RIPA buffer for 20 minutes, then the cells were collected with cell scraping, and centrifuged to obtain cell pellet.

### 2.3 Plasmid transfection

GC cells (5 × 10^5^) were seeded into six‐well plates with antibiotic‐free medium containing 3 µg plasmid and 5 µL LiP3000TM dissolved in 125 µL Opti‐MEM. Then, 7.5 µL Lipofectamine 3000 reagent (Invitrogen) was added to another 125 µL Opti‐MEM, mixed gently with the two 125 µL Opti‐mixed MEM and incubated for 5 minutes at room temperature. Finally, the mixture was placed into a six‐well plate, gently blended, and then placed into the incubator.

### 2.4 Methylation bisulfite sequencing PCR (BSP) experiment

Genomic DNA was extracted from cells transfected with control αSR and experimental CagA plasmids for 48 hours, and the genomic DNA was bisulfite converted. The primers were designed for the KLF4 promoter region, the primers were used for PCR amplification, and the amplification products were sequenced.

### 2.5 Western blotting

GES‐1 and gastric cancer cells were seeded into six‐well plates at 5 × 10^5^ cells/well and incubated for 24 hours. Cells were co‐cultured with *H pylori* or transfected with plasmids for corresponding time. Cells were harvested and lysed using RIPA buffer. Cellular proteins were collected, degraded, and subjected to sodium dodecyl sulfate‐PAGE (SDS‐PAGE) electrophoresis. Proteins were transferred to polyvinylidene fluoride membranes (PVDF membranes, Millipore). Then, the membranes were blocked for 1 hour and incubated with primary antibodies KLF4 (Santa Cruz), TET1 (Santa Cruz), HA (OriGene), and β‐catenin (Cell Signaling Technology) overnight at 4°C. The membranes were washed and incubated with secondary antibodies for 1 hour at room temperature. Finally, the resultant bands were detected using an Odyssey Infrared Imaging system (LI‐COR, NB, USA).

### 2.6 Reverse transcription‐PCR (RT‐PCR)

GES‐1 and gastric cancer cells were seeded into six‐well plates at 5 × 10^5^ cells/well and incubated for 24 hours. Cells were co‐cultured with *H pylori* or transfected with plasmids for corresponding time. The cells were collected and lysed. Chloroform and isopropanol were added to obtain RNA precipitation and then washed with 75% alcohol to obtain genomic RNA of the template. The concentration and quality of the RNA is qualified, then follow the reverse transcription procedures: template RNA 3 μg, oligo 1 µL, Rnase‐free water supplementation to 12 µL, metal bath at 65°C for 5 minutes, placed on ice; Adding 5× Reaction Buffer 4 µL, RTM RNA enzyme inhibitor 1 µL, 10 mmol/L dNTP Mix 2 µL, and RTM reverse transcriptase (10µ/ µL) 1µL, mixed them gently, centrifuge them at low speed for several seconds, and place them in a PCR instrument for 42° 60 minutes, 70° 5 minutes, Finally got template DNA. RT‐PCR was performed according to standard procedures: 12.5 µL 2× Es Taq Master Mix, 1 µL 10 µmol/L forward primer, 1 µL 10 µmol/L reverse primer, 8.5 µL RNase‐free water, and 2 µL template DNA were mixed gently in the tube. The primers (Invitrogen) for the RT‐PCR assay are indicated in Table S1. DNA Ladder and PCR product 10 µL were successively added into the 1.5% agarose gel, filled with electrophoretic fluid, maintained the pressure 90 v for 30 minutes until bromophenol blue ran through 2/3 of the gel. The resultant bands were detected using an Odyssey Infrared Imaging system (LI‐COR, NB, USA).

### 2.7 Colony formation assay

Five hundred GC cells and normal gastric epithelial cells were transiently transfected with the corresponding plasmid for 24 hours in a six‐well plate, 2 mL of medium was added, and the cells were gently placed in a 37°C incubator for 2 weeks. Then, the cells were washed with PBS in six‐well plates, fixed for 20 minutes, and dyed for 30 minutes with crystal violet. The colonies were counted with the naked eye and photographed.

### 2.8 Methyl thiazolyl tetrazolium (MTT) assay

GC cells and normal gastric epithelial cells (3 × 103) were transiently transfected with the corresponding plasmid for 24 hours. An MTT assay measured the cell viability at 0, 24, 48, and 72 hours.

### 2.9 Cell migration assays

Counted cells (7 × 10^4^) were placed into transwell (Corning) membranes for 24 hours. The top chamber was filled with serum‐free growth medium, while the bottom chamber was filled with medium supplemented with 5% serum. Then, the cells were washed gently with PBS, fixed for 15 minutes at room temperature, and dyed with crystal violet for 30 minutes. Next, the transwell membranes were gently washed with water, and air‐dried. Finally, the number of cells was directly counted and photographed.

### 2.10 Immunohistochemical experiment

Eighty‐eight cases of tissue adjacent to carcinoma and GC tissue were cut into 3 µL sections and then deparaffinized. Antigen retrieval was performed with citric acid buffer. Slides were incubated with primary antibodies against KLF4 (Santa Cruz) and 5‐hmC (Active Motif) overnight at 4°C. The positive brown granular substances were detected and a result score was given based on the percentage of positive cells and the dyeing depth of KLF4 and 5‐hmC. Each section was evaluated by two pathologists double‐blinded in randomized observations with 10 high‐power fields of vision. The positive cell percentage points were as follows: >75% for 4 points, 50%‐75% for 3 points, 25%‐50% for 2 points, 10%‐25% for 1 point, <10% for 0 points. Positive staining intensity was defined as follows: colorless meter for 0 points, pale yellow meter for 1 point, tan for 2 points, and tan meter for 3 points. Both scoring systems indicate the expression level: strong positive: >6 points; weak positive: 3‐6 points; negative: ≤3 points.

### 2.11 Statistical analysis

All data were presented as the mean ± standard deviation from at least three independent experiments. Unless otherwise noted, the differences between groups were analyzed using Student's *t* test for two groups or assessed by one‐way analysis of variance (ANOVA) when more than two groups were compared. All tests performed were two‐sided. In clinicopathological parameters analyzation, Pearson's chi‐square test was performed for statistical analyses. *P < .05, **P < .01, ***P < .001.

## 3 RESULTS

### 3.1 Reduction in or loss of KLF4 expression in GC

**Figure 1:**
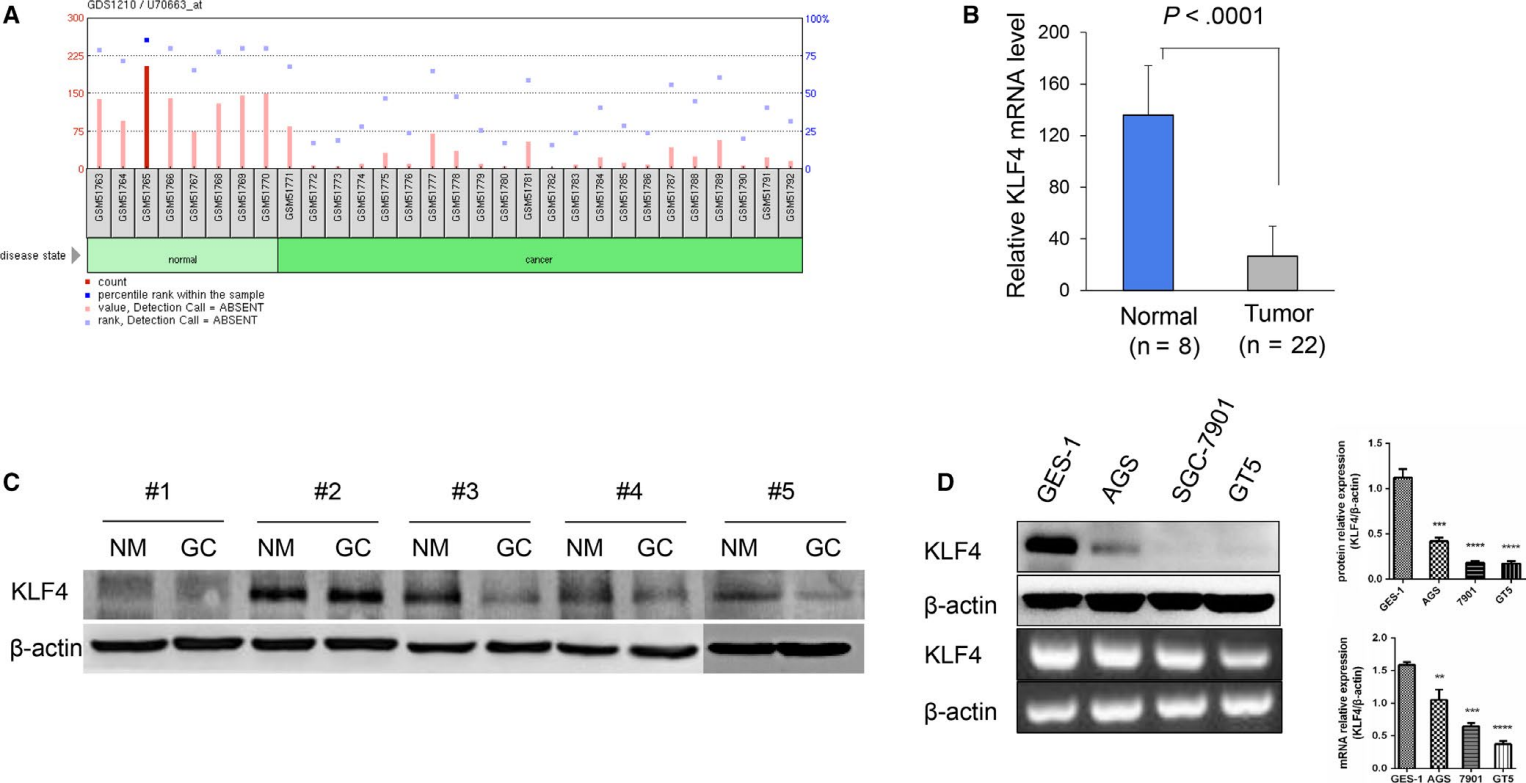
Reduced or loss of KLF4 expression in gastric cancer. A and B, mRNA expression of KLF4 drastic reduce in gastric cancer tissues compare to normal gastric tissues from GEO profiles:GDS1210. C, The protein expression of KLF4 is decreased by western blot analysis in gastric cancer tissues of surgery patients. D, The mRNA and protein expression of KLF4 in 4 cell lines by RT‐PCR and western blot analysis. The experiments were performed independently three times. Data are shown as mean ± SD. *P < .05,**P < .01

High expression of KLF4 in the normal gastric epithelium and low expression or loss has been demonstrated often in GC tissues.^6^ From the GEO profile GDS1210, we found a drastic reduction in the expression of KLF4 mRNA in GC compared with tissues from the disease state (Figure 1A,B). In addition, we collected five groups of normal stomach and GC tissue from clinical GC surgical patients and showed that compared with normal tissues, GC tissues had significantly reduced KLF4 protein expression (Figure 1C). Moreover KLF4 expression was lower in GC cells (GT5, SGC7901, and AGS) than in normal gastric epithelial cells (Figure 1D). Collectively, these data illustrated that KLF4 expression was decreased in GC.

### 3.2 *H pylori* infection or CagA transfection resulted in a reduction in or loss of KLF4 expression in GC cells

**Figure 2:**
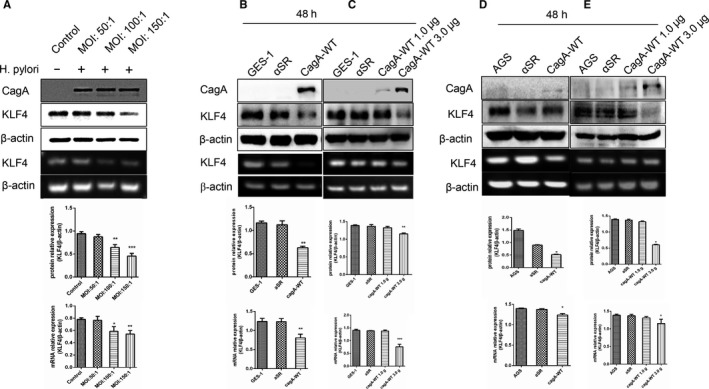
*Helicobacter pylori* infection or CagA transfection lead to reduced or loss of KLF4 expression. A, GES‐1 cells were co‐cultured with different concentrations of *H pylori* (50:1,100:1,150:1) for 48 h, then KLF4 mRNA and protein level of GES‐1 cell significantly decrease. B and D, WB and RT – RCR found that the GES‐1 and AGS transiently transfected CagA 3.0 μg for 48 h, and KLF4 (mRNA and protein) expression is decreased obviously. C, When GES‐1cells transfected CagA 1.0 μg for 48 h, KLF4 mRNA and protein had no change; transfection dose increases to 3.0 μg for 48 h, KLF4 (mRNA and protein) expression significantly decreased by WB and RT‐PCR. E, When AGS cell transfected CagA 1.5 μg for 48 h, KLF4 expression had no change; transfection dose increases to 3.0 μg for 48 h, KLF4 (mRNA and protein) expression significantly reduce. The experiments were performed independently three times

Tumor incidence is linked to gene regulation and is also affected by environmental factors. *H pylori* has been defined as the first kind of biological carcinogenic factor in GC.^18^, ^19^, ^20^ However, KLF4 plays a role as a tumor suppressor gene in GC. Previous studies reported that *H pylori* CagA promotes malignant transformation of gastric epithelial cells through the dysregulation of mir‐155/KLF4 signaling pathway.^14^ To explore the molecular mechanisms between KLF4 and either *H pylori* infection or CagA, we chose different concentrations of *H pylori* for coculture with GES‐1 cells for 48 hours, and then the RNA and protein were collected. The results showed that KLF4 expression significantly decreased at the RNA and protein levels with an increase in the dose of *H pylori*‐infected GES‐1 cells and an increase in the amount of CagA protein in cells (Figure 2A). We chose GES‐1 cells with high KLF4 expression and GC AGS cells with relatively high KLF4 expression and transiently transfected these cells with plasmids containing αSR (3.0 µg) and the CagA‐wt virulence protein (3.0 µg), respectively, for 48 hours. Western blot experiments detected that *H pylori* CagA gene transduction resulted in significantly reduced KLF4 protein expression in GES‐1 and AGS cells. On the one hand, RT‐PCR experiments detected that KLF4 gene expression was also significantly decreased (Figure 2B,2). On the other hand, when the GES‐1/AGS cells were transfected with different dose of CagA plasmid, we found that KLF4 (mRNA and protein) expression is no change for 1.0/1.5 μg CagA plasmid. While, KLF4 (mRNA and protein) expression significantly decreased for 3.0 μg CagA plasmid compared with control (Figure 2C,E). Therefore, in both GES‐1 and AGS cells, the induced expression of CagA resulted in significantly reduced KLF4 expression of tumor suppressor genes.

### 3.3 Induced CagA expression in GES‐1 and AGS cells promoted behavioral change

**Figure 3:**
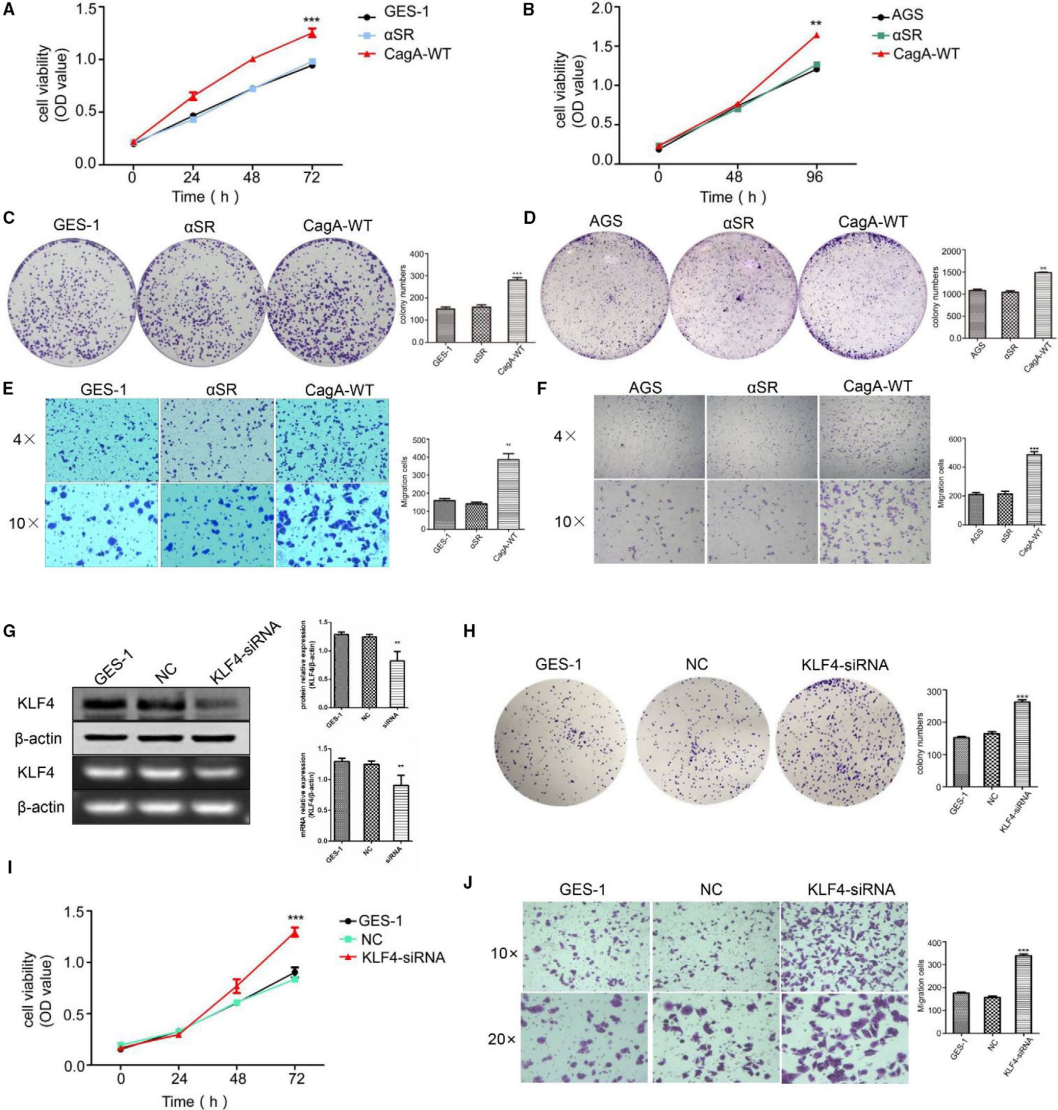
Enforced expression of CagA in GES‐1 and AGS cells promoted behavioral change. A and B, When GES‐1 and AGS cells transfected CagA for 24 h, GES‐1 cells(0, 24, 48, 72 h) and AGS cells(0, 48, 96 h) proliferation significantly enhance by MTT assays. C and D, When GES‐1 and AGS cells transfected CagA for 24 h, cell proliferation significantly enhance after two weeks by clone formation assays. E and F, When GES‐1 and AGS cells transfected CagA for 24 h, cell migration significantly enhance after 36 h by transwell assays. G, When GES‐1 cell transfected KLF4 siRNA for 48 h, the protein and mRNA level is obviously reduced. H, I, and J, When GES‐1 cell transfected KLF4 siRNA for 48 h, the cell ability of proliferation, clone formation and migration is significantly increased. The experiments were performed independently three times

Similar to a large number of studies, we found that KLF4 is related to the regulation of cell differentiation and proliferation.^21^, ^22^, ^23^, ^24^, ^25^ Therefore, the GC cells AGS and the normal gastric epithelial cells GES‐1 were transiently transfected with the empty vector plasmid αSR and the plasmid with *H pylori* CagA‐wt (3.0 µg), respectively, and then the cell proliferation and migration were detected. The MTT assay found that when CagA‐wt was overexpressed, the proliferation abilities of GES‐1 and AGS cells were significantly enhanced (Figure 3A,B). In addition, the same results were found in both colony formation assays (Figure 3C,D) and cell migration assays (Figure 3E,F).

To further validate that induced CagA protein expression promotes cell behavior changes by inhibiting the expression of KLF4, we designed KLF4 siRNA and then transiently transfected the siRNA into a normal gastric epithelial GES‐1 cell line to knockdown KLF4 expression. Behavioral changes were the same: the enhanced proliferation, clone formation, and migration abilities were similar in transfected KLF4 siRNA GES‐1 cells as in transfected CagA GES‐1/AGS cells (Figure 3G,H,I,J).

### 3.4 CagA transfection increases KLF4 promoter methylation

**Figure 4:**
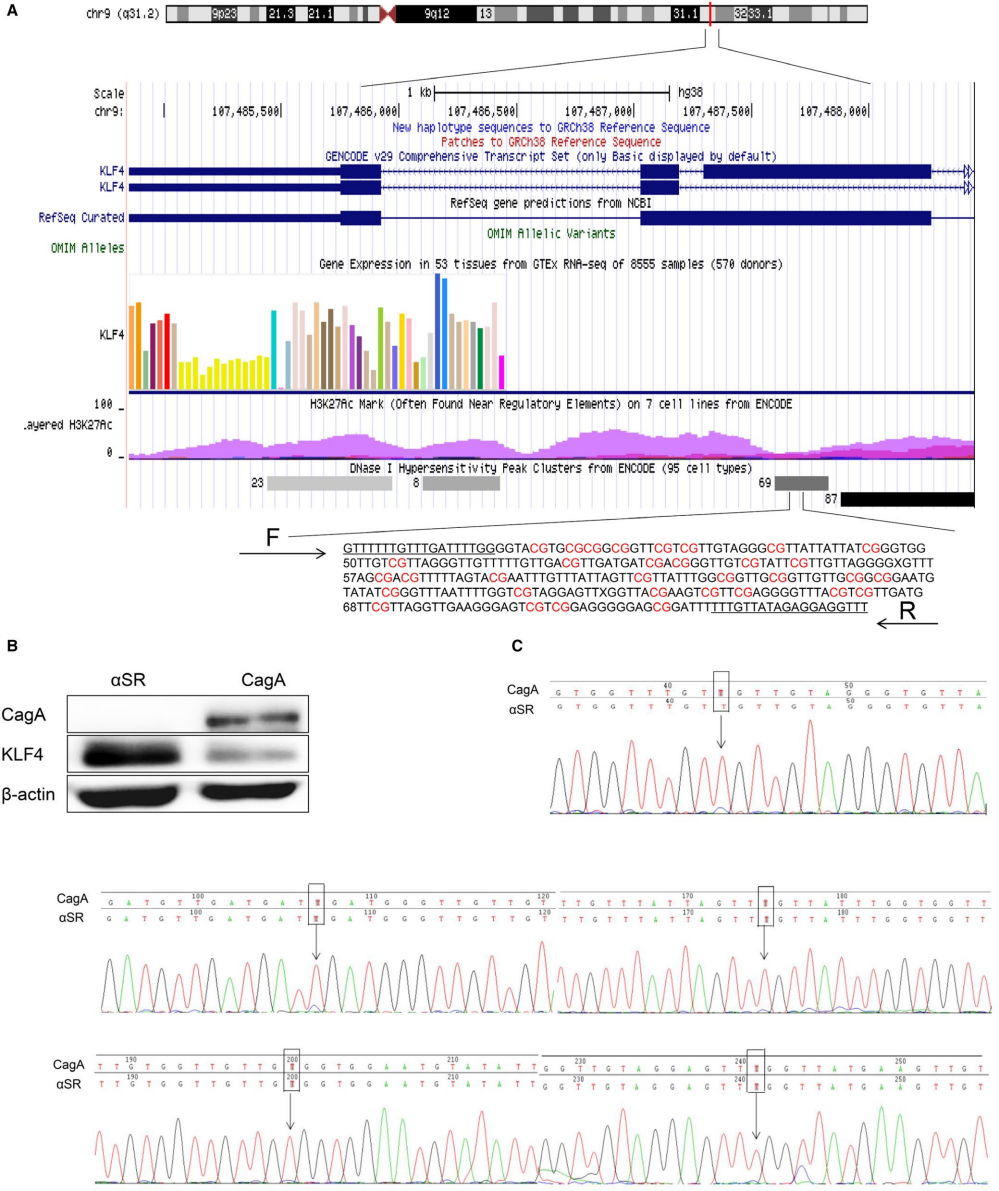
Genetic and epigenetic alterations of KLF4 in GES‐1 cells transfected CagA for 48 h. A, Diagram of CpG islands and sequence in the promoter region of KLF4 gene. B and C, Bisulfite‐DNA sequence analysis confirmed the existence of KLF4 promoter methylation in GES‐1 cells transfected CagA. Note: * indicates methylated C in CpG sites

It has been reported that KLF4 expression was reduced in a series of GC tissue and GC cell lines could be due to promoter methylation,^17^ but *H pylori* infection increased the promoter methylation of tumor suppressor genes.^26^, ^27^, ^28^ To explore whether CagA increases the KLF4 promoter methylation levels to reduce KLF4 expression, we analyzed the CpG island methylation level of the KLF4 promoter region from the transcription start site to the first exon (Figure 4A). The genomic DNA of GES‐1 cells was transiently transfected with CagA for 48 hours, while western blotting showed that KLF4 protein expression decreased (Figure 4B). After bisulfite treatment of genomic DNA, BSP primers were used to amplify the fragment; we found that due to transfection efficiency, the KLF4 promoter CpG island methylation was slightly higher in the CagA plasmid transfection group than in the control group (Figure 4C).

### 3.5 CagA inhibits TET1 protein expression to reduce KLF4 expression

**Figure 5:**
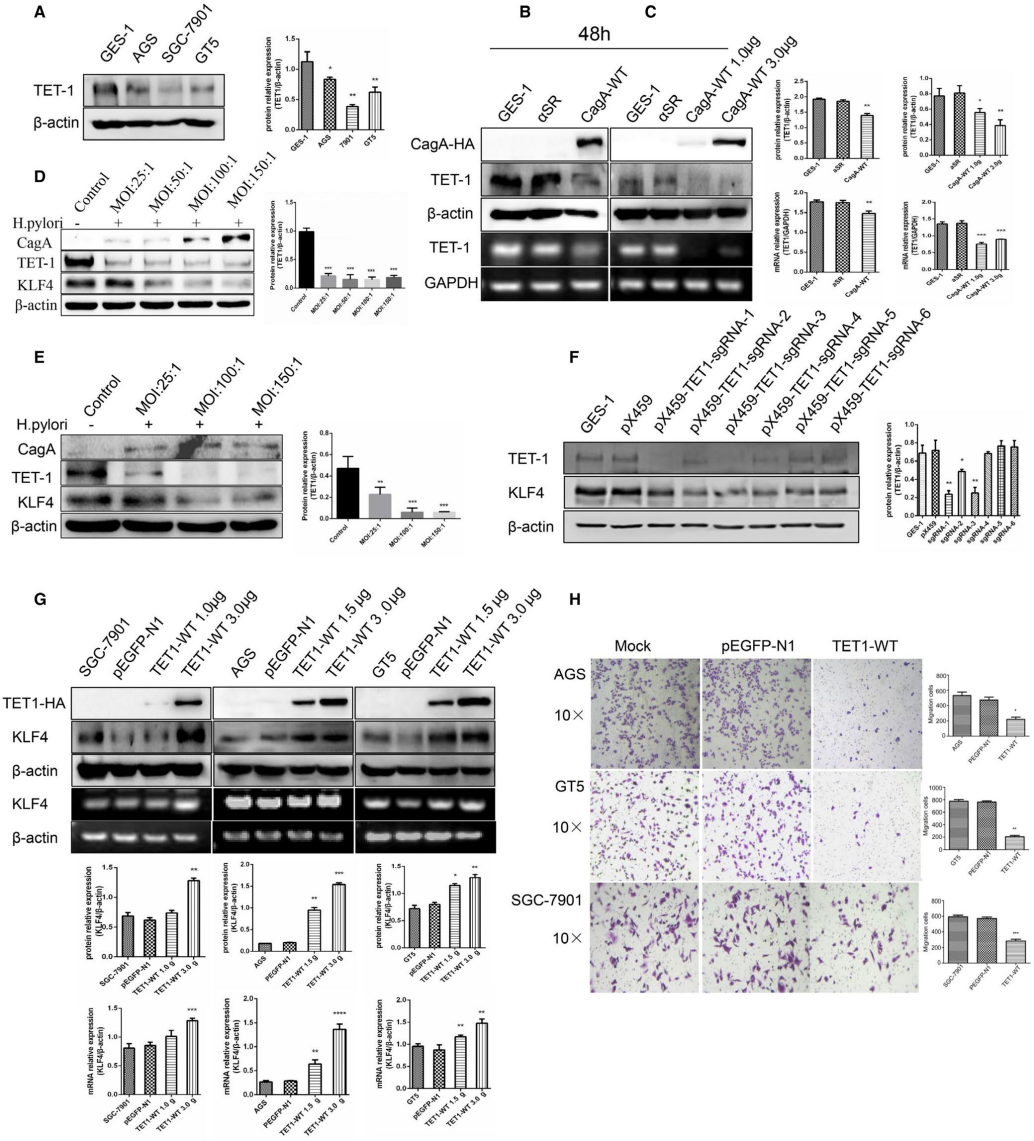
CagA inhibits protein TET1 to reduce KLF4 expression. A, TET1 protein is low expression in gastric cancer cell lines compare to GES‐1 cell by Western blot assay. B, When GES‐1 cell transient transfected CagA 3.0 μg for 48 h, TET1 expression significantly decreased. C, In dose‐response experiment, when GES‐1 cell transfected CagA 1.0 μg for 48 h, TET1 expression reduced; when the transfection dose increases to 3.0 μg for 48 h, TET1 expression significantly decreased. D, GES‐1 cells were co‐cultured with different concentrations of *H pylori* (25:1,50:1,100:1,150:1) for 48 h, then CagA protein expression gradually increased and KLF4 protein expression gradually reduced, while TET1 protein expression obviously reduce. E, AGS cells were co‐cultured with different concentrations of *H pylori* (25:1, 100:1,150:1) for 48 h, then CagA protein expression increased and TET1 and KLF4 protein expression gradually reduce. F, Construction of plasmid with CRISPR/Cas9 technology to knockdown TET1 in GES‐1 cell, KLF4 expression is decreased obviously then choose a stable cell line. G, AGS, SGC‐7901, GT5 cell transfected TET1 with 1.0 μg or 1.5 μg and 3.0 μg for 48 h, KLF4 expression gradually increase. H, AGS, SGC‐7901, GT5 cell transfected TET1 3.0 μg for 48 h, then migration ability decreased obviously. The experiments were performed independently three times

Gene methylation and demethylation are dynamic processes. TETs are a protein family of DNA demethylation proteins whose main functions include converting 5‐mC into 5‐hmC and participating in the process of cell genome demethylation.^29^ It has been reported that TET1 is positively correlated with 5‐hmC and is reduced in GC, while TET2 and TET3 mRNA are not different in GC.^30^ We also found that TET1 protein expression was lower in GC cells than in GES‐1 cells (Figure 5A). Therefore, we speculated that *H pylori* coculture affects TET1 expression in normal gastric epithelial cells, which influences the genome methylation level and ultimately leads to reduced KLF4 expression. Similarly, we examined the TET1 changes after CagA overexpression in GES‐1 cells. GES‐1 cells were transiently transfected with CagA for 48 hours, and we found that TET1 expression were reduced (Figure 5B). Moreover GES‐1 cells transfected with the CagA plasmid at 1.0 µg and 3 µg caused the expression of TET1 to gradually decrease (Figure 5C). To further investigate this, we chose different concentrations of *H pylori* for coculture with AGS and GES‐1 cells for 48 hours, which showed that with the increase in *H pylori* infection dose and, KLF4 and TET1 expression gradually decreased (Figure 5D,E). We also used CRISPR/Cas9 gene editing techniques to construct the plasmid pX459 with TET1‐sgRNA, which was transfected into GES‐1 cells to knockdown expression of TET1, and stable cell lines were established. Western blotting showed that KLF4 expression was significantly decreased when TET1 was knocked down with CRISPR/Cas9 gene editing techniques (Figure 5F). In this study, we chose SGC‐7901, AGS, and GT5 cells, with lower TET1 expression levels; these cells were transiently transfected with plasmids containing the empty vector, pEGFP‐N1, and TET1‐ wt (1.0 µg or 1.5 µg and 3 µg) for 48 hours, and KLF4 expression significantly increased (Figure 5G). We also measured the changes in cell migration ability when the three GC cell lines overexpressed TET1. Compared with cells transfected with the empty vector, SGC‐7901, AGS, and GT5 cells transiently transfected with the TET1‐wt plasmid for 24 hours showed significantly reduced migration (Figure 5H). In addition, we selected the stable cell lines of GES‐1‐transfected pX459 and pX459 TET1‐sgRNA (Figure S1A), extracted genomic DNA, detected the methylation level of the KLF4 promoter regions of the CpG island, and found that the KLF4 promoter region methylation level was slightly higher in the transfected cells than in the control cells (Figure S1B). Therefore, KLF4 expression was significantly enhanced when TET1 was overexpressed.

### 3.6 KLF4 expression is lower in GC tissues with *H pylori* infection than in GC tissues without *H pylori* infection

We collected all of the clinical data from 88 GC patients who had an *H pylori* examination at the First Affiliated Hospital of Anhui Medical University in 2013. Using a chi‐square test, we found that *H pylori* infection, lymph node metastasis, and the stomach tissue differentiation type had statistical significance, which means that positive *H pylori* infections in GC patients promote lymph node metastasis and poor differentiation (Table 1).

**Table 1:** Analysis of tumor characteristics and HP infection in the 88 gastric cancer patients

Characteristic	Total (n = 88)	HP^+^ (%) n = 61	HP^‐^ (%) n = 27	*P*
Sex				
Man	67	44 (46.4)	23 (20.6)	.185
Woman	21	17 (14.6)	4 (6.4)	
Age (y)				
<65	57	40 (39.5)	17 (17.5)	.813
≥65	31	21 (21.5)	10 (9.5)	
Tumor size (cm)				
<5	44	30 (30.5)	14 (13.5)	.817
≥5	44	31 (30.5)	13 (13.5)	
Pathology type				
Papillary	15	11 (10.4)	4 (4.6)	.068
Tubular	25	20 (17.3)	5 (7.7)	
Diffuse	11	4 (7.6)	7 (3.4)	
Mucinous	17	10 (11.8)	7 (5.2)	
Signet ring	5	3 (3.5)	2 (1.5)	
Mixed	15	13 (10.4)	2 (4.6)	
Depth of tumor invasion				
T1	13	6 (9.0)	7 (4.0)	.175
T2	27	19 (18.7)	8 (8.3)	
T3	30	21 (20.8)	9 (9.2)	
T4	18	15 (12.5)	3 (5.5)	
Metastatic lymphnodes				
N0	28	16 (19,4)	12 (8.6)	.048
N1	15	8 (10.4)	7 (4.6)	
N2	27	21 (18.7)	6 (8.3)	
N3	18	16 (12.5)	2 (5.5)	
Stage				
Ⅰ	24	13 (16.5)	11 (7.4)	.118
Ⅱ	8	7 (5.5)	1 (2.5)	
Ⅲ	37	25 (25.6)	12 (11.4)	
Ⅳ	19	16 (13.2)	3 (5.8)	
Histological type				
Well diff	8	2 (5.5)	6 (2.5)	.000
Moderately diff	22	11 (15.3)	11 (6.8)	
Poorly diff	58	48 (40.2)	10 (17.8)	

Note

Pearson's χ^2^ test was done to determine the statistical significance of the relationship of HP infection with various variables. As the coloured values show, H pylori infection positively correlation with lymph node metastasis (*P* = .048) and the stomach tissue differentiation type (*P* = .000), and had statistical significance

*P* < .05.

**Figure 6:**
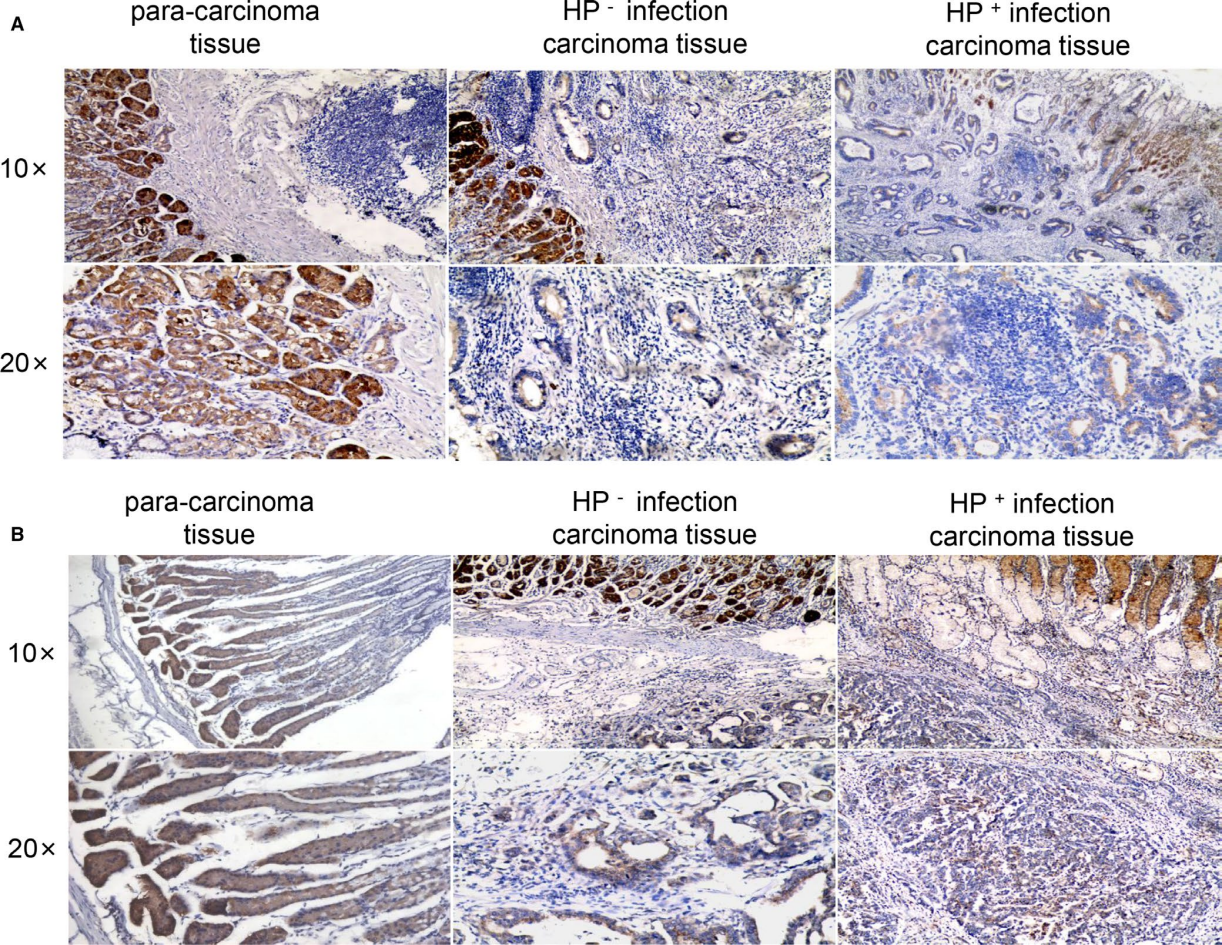
KLF4 and 5‐hmc expression in tissues of adjacent to carcinoma, *Helicobacter pylori* negative infection gastric cancer, *H pylori* positive infection gastric cancer determined by IHC analysis. A, KLF4 expression in tissues of adjacent to carcinoma, *H pylori* negative infection gastric cancer, *H pylori* positive infection gastric cancer is gradually reduced. B, 5‐hmc expression in tissues of adjacent to carcinoma, *H pylori* negative infection gastric cancer, *H pylori* positive infection gastric cancer is gradually reduced

Immunohistochemical experiments found that KLF4 expression was significantly increased in the adjacent normal tissues compared to that in the carcinoma tissue and was significantly decreased in GC patients with positive *H pylori* infection compared with expression in those without *H pylori* infection (Figure 6A). Similarly, 5‐hmC expression in adjacent normal tissues was much stronger than that in the carcinoma tissue, and 5‐hmC expression was increased in the GC tissue of GC patients without *H pylori* infection compared to that in the GC tissue of *H pylori*‐positive GC patients (Figure 6B). Finally, we obtained the results of immunohistochemical analysis of KLF4 and 5‐hmC (Table 2). The results show that in GC patients without *H pylori* infection, KLF4 expression has a strong positive rate of 51.9% in GC tissue, while in GC patients with *H pylori* infection, KLF4 expression has a positive rate of 27.9% in GC tissue; these results indicate that expression of KLF4 is apparently decreased in *H pylori*‐infected patients compared to that in patients without *H pylori* infection (*P* = .042). Similarly, the strong positive rate of 5‐hmC expression was 51.9% in GC tissue without *H pylori* infection, while that in GC tissues of patients with *H pylori* infection was 26.2% (*P* = .020).

**Table 2:** *Helicobacter pylori* infection was associated with the expression of KLF4 and 5hmC in gastric cancer tissues

	Total	Negative	Weak	Strong	*P*	*χ^2^*
KLF4 expression						
HP(+)	61 (61.0)	26 (42.6%)	18 (29.5%)	17 (27.9%)	0.042	6.143
HP(−)	27 (27.0)	5 (18.5%)	8 (29.6%)	14 (51.9%)		
5hmc expression						
HP(+)	61 (61.0)	25 (41.0%)	20 (32.8%)	16 (26.2%)	0.020	7.495
HP(−)	27 (27.0)	4 (14.8%)	9 (33.3%)	14 (51.9%)		

Note

HP(+) *Helicobacter. Pylori* infection positive, HP(−) *Helicobacter. Pylori* infection negative.

## 4 DISCUSSION

In stomach cancer, lower KLF4 expression is related to patient prognosis and lymph node and distant metastases. A functional experimental study showed that KLF4 overexpression significantly promoted apoptosis and inhibited cell cycle progression in vitro, and reduced the tumorigenicity of GC in vivo.^31^, ^32^ Besides, KLF4 inhibits cell proliferation, promotes cell differentiation and is closely related to tumor development.^21^, ^22^ Targeting KLF4 in advanced solid tumors has been approved for clinical trials.^7^ In this study, we show that KLF4 expression is lower in both GC patient’ samples and cell lines compare to controls. However, the molecular mechanism of KLF4 inactivation in GC is still unclear and needs to be further elucidated.

The formation of tumors is regulated by genes and environmental factors.^33^Most infected people have superficial gastritis, which means that the organism plays a causative role in early tumorigenesis.^34^, ^35^, ^36^ Epidemiology and clinical studies have found that the effective treatment and eradication of *H pylori* can reduce GC incidence.^37^ *H pylori* infection could aggravate the prognosis of GC: compared to early GC patients treated for *H pylori*, patients treated with the placebo had a decreased chance of heterotopic GC, and their baseline classification of gastric atrophy improved.^38^ A large amount of epidemiological data also confirmed that patients infected with CagA+ *H pylori* have a much stronger risk of developing GC than patients who are free of CagA pylorus helix bacillus infection.^39^ CagA is the main virulence factor, which is delivered into gastric epithelial cells with the help of the T4SS system after tyrosine phosphorylation by the Src family kinases^40^; phosphorylated CagA specifically binds to Shp‐2 and aberrantly activates it. CagA could interact with or inhibit microtubule affinity‐regulating kinase (MARK)/ partitioning‐defective 1 (PAR1), leading to junctional and polarity defects in epithelial cells, independent of CagA tyrosine phosphorylation.^41^, ^42^, ^43^ Moreover CagA transgenic expression of *H pylori* induced tumors in the gastrointestinal tract and hematopoietic mice.^44^ We have reported that *H pylori* CagA promotes malignant transformation of gastric epithelial cells through the dysregulation of mir‐155/KLF4 signaling pathway.^14^ In addition, our data also suggest that *H pylori/* CagA reduce the KLF4 expression and increase the ability of proliferation, clone formation, and migration in GES‐1 and AGS cellsKLF4 promoter methylation is an important cause of GC due to its reduced expression in GC.^6^, ^45^ DNA methylation and demethylation are dynamic processes mediated by the DNMT and TET family proteins, respectively. The most important function of TET1 (ten‐eleven translocation) protease activity is to convert 5‐methylcytosine (5‐mC) into 5‐hydroxymethylcytosine (5‐hmC), while the TET2 and TET3 mRNAs do not differ in GC.^29^, ^30^, ^45^, ^46^, ^47^, ^48^, ^49^ Tet1 expression was also significantly reduced in CagA + GC tissues compared with that in noncancerous tissues.^18^ According to many studies and experimental data, *H pylori* infection/CagA promotes the transport of the DNMT1 and DNMT3B proteins (DNA methyltransferase 3 B) into the nucleus of cells, and this may be related to the silencing of transcriptional expression, such as E‐cadherin (CDH1) and forkhead box (Fox).^50^, ^51^ On the one hand, by analyzing genome DNA sequencing data, the methylation level of the KLF4 promoter slightly increased in GES‐1 cells transiently transfected with the CagA plasmid compared with that in control cells. On the other hand, we found that GES‐1/AGS transient transfected CagA plasmid/ infected *H pylori* inhibits TET1 expression, and TET1 further reduced KLF4 expression. These results suggest that CagA inhibits TET1 expression, which increases the methylation level of the KLF4 promoter, causing KLF4 expression to decrease.

In summary, we revealed that the CagA protein and *H pylori* infection inhibit KLF4 expression by downregulating TET1 expression and affecting KLF4 promoter methylation. These novel findings not only help us to further study the molecular pathogenesis of GC but also provide a novel molecular target of CagA‐TET1‐KLF4 signaling for GC treatment.

## CONFLICT OF INTEREST

The authors declare no potential conflicts of interest.
