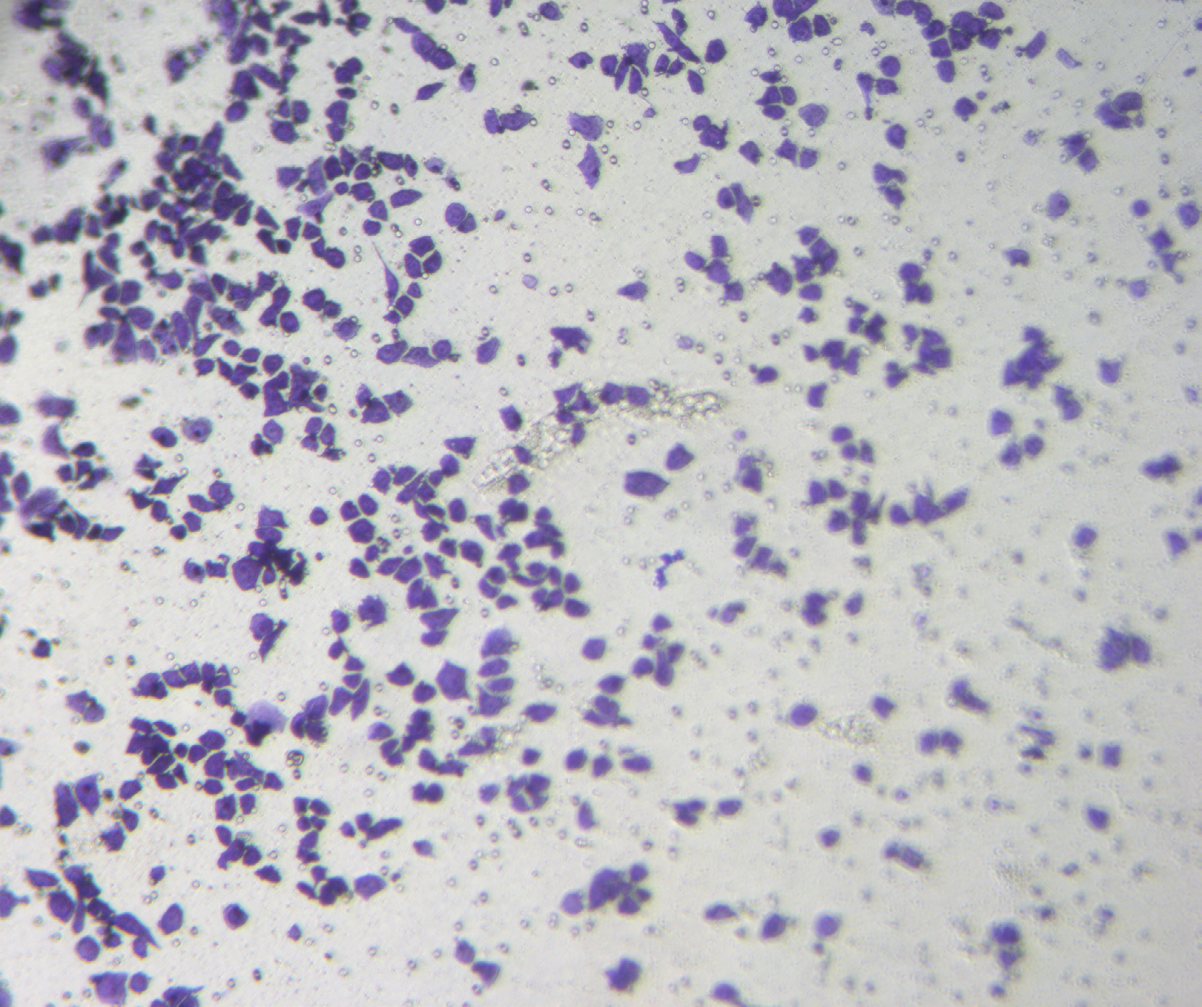# Correction to *Helicobacter pylori* infection leads to KLF4 inactivation in gastric cancer through a TET1‐mediated DNA methylation mechanismZhao, R, Liu, Z, Xu, W, Song L., Ren H., Ou Y., Liu Y., Wang S. *Helicobacter pylori* infection leads to KLF4 inactivation in gastric cancer through a TET1‐mediated DNA methylation mechanism. Cancer Med 2020; 9: 2551–2563. https://doi.org/10.1002/cam4.2892

**DOI:** 10.1002/cam4.5878

**Published:** 2023-06-12

In Figure 5H a duplication in the images was detected due to an error by the author group. The corrected panel is shown below.

The authors regret this error.