# Supplementary figures and images for: Helicobacter pylori infection leads to KLF4 inactivation in gastric cancer through a TET1‐mediated DNA methylation mechanism
Source: Cancer Med. 2020 Feb 4;9(7):2551–63. doi: 10.1002/cam4.2892 (PMC7131848; doi:10.1002/cam4.2892)

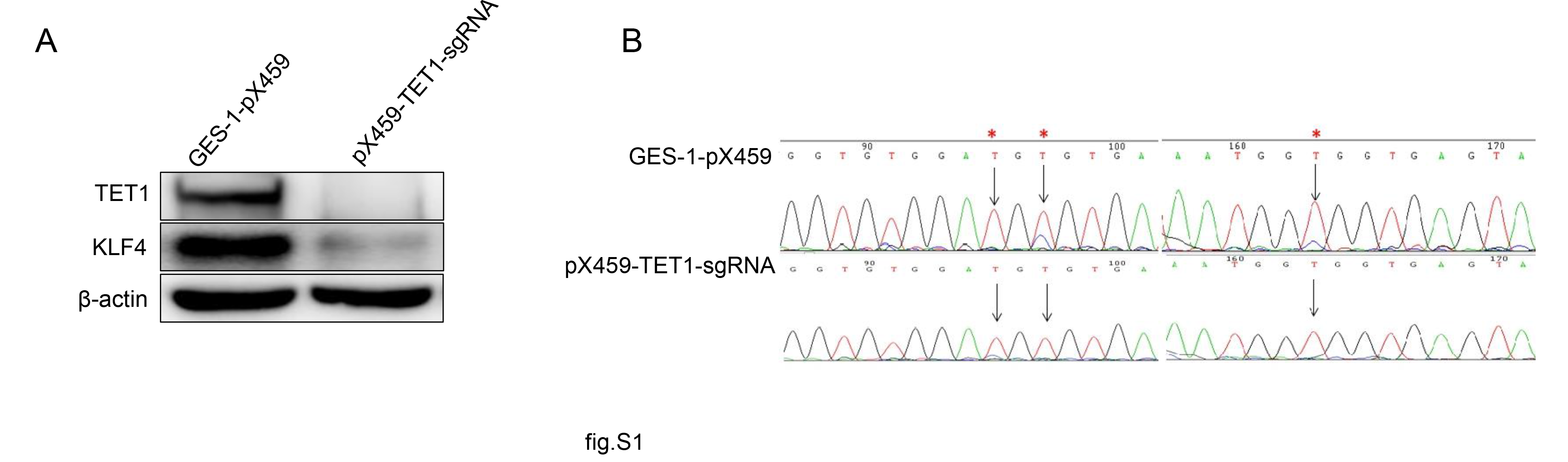

Supplement: Supplementary file 1 [file CAM4-9-2551-s001.tif]
