# Supplementary material for: Helicobacter pylori infection leads to KLF4 inactivation in gastric cancer through a TET1‐mediated DNA methylation mechanism
Source: Cancer Med. 2020 Feb 4;9(7):2551–63. doi: 10.1002/cam4.2892 (PMC7131848; doi:10.1002/cam4.2892)
Supplement: Supplementary file 2 [file CAM4-9-2551-s002.docx]

| **Gene name** | **Forward primer (5’-3’)** | **Reverse primer (5’-3’)** |
| --- | --- | --- |
| **KLF4** | GCTGGACCCCCTCTCAGCAATGG | AGAAAATCTGGCACCACACC |
| **β-actin** | ATCACAAGTGGGTGGCGGTCC | CTCCTTAATGTCACGCACGA |
